# Collection of population-based cancer staging information in Western Australia – a feasibility study

**DOI:** 10.1186/1478-7954-3-9

**Published:** 2005-08-17

**Authors:** Timothy Threlfall, Jana Wittorff, Padabphet Boutdara, Jane Heyworth, Paul Katris, Harry Sheiner, Lin Fritschi

**Affiliations:** 1 Western Australian Cancer Registry, Department of Health, Government of Western Australia, Perth, Australia; 2 School of Population Health, University of Western Australia, Perth, Australia; 3 Western Australian Clinical Oncology Group, The Cancer Council of Western Australia, Perth, Australia

## Abstract

**Background:**

Routine data from cancer registries often lack information on stage of cancer, limiting their use. This study aimed to determine whether or not it is feasible to add cancer staging data to the routine data collections of a population-based Western Australian Cancer Registry (WACR).

**Methods:**

For each of the five most common cancer types (prostate, colorectal, melanoma, breast and lung cancers), 60 cases were selected for staging. For the 15 next most common cancer types, 20 cases were selected. Four sources for collecting staging data were used in the following order: the WACR, the hospital based cancer registries (HBCRs), hospital medical records, and letters to treating doctors. If the case was unable to be fully staged, due to lack of information on regional lymph node invasion or distant metastases, we made the following assumptions. Cases which had data available for tumour (T) and regional lymph nodes (N), but no assessment of distant metastasis (MX) were assumed to have no distant metastases (M0). Cases which had data for T and M, but no assessment of regional nodal involvement (NX) were assumed to have no regional nodal involvement (N0).

**Results:**

The main focus of this project was the process of collecting staging data, and not the outcomes. For ovary, cervix and uterus cancers the existence of a HBCR increased the stageable proportion of cases so that staging data for these cancers could be incorporated into the WACR immediately. Breast and colorectal cancer could also be staged with adequate completeness if it were assumed that MX = M0. Similarly, melanoma and prostate cancer could be staged adequately if it were assumed that NX = N0 and MX = M0. Some cases of stomach, lung, pancreas, thyroid, testis and kidney cancers could be staged, but additional clinical input – on pathology request forms, for example – would be required to achieve useable levels of completeness. For the remaining cancer types either staging is widely regarded as not relevant, and no generally-accepted system exists, or an acceptable level of completeness is not achievable.

**Conclusion:**

Adding stage to routinely collected information in a cancer registry is possible for many cancer types, particularly if the assumptions regarding missing data are found to be acceptable or if the guidelines for MX = M0 asumptions are clarified. These findings should be generalizable to most cancer registries in developed countries, if hospital-based cancer registries or other specialized databases are accessible.

## Background

Cancer staging information is of fundamental importance at both population and individual levels. For the individual, it facilitates provision of appropriate patient care, enables appropriate selection of treatment for individual cases, can be used to explain variability in treatment outcomes, and can contribute to helping an individual patient and their family to better understand the clinical condition and prognosis. At the population level, staging data can guide the development and evaluation of health promotion and treatment programs, can facilitate more effective resource allocation depending on the relative proportions of "early" as opposed to "late" cases and can be used to stratify survival analyses to improve comparison of results of different groups such as geographic areas [1-3]. The lack of staging data has been recognised by clinicians and public health professionals alike as an important limitation of cancer registry data.

Different staging systems exist and they have different rules and guidelines. Commonly used staging systems include those of the American Joint Committee on Cancer (AJCC), International Union Against Cancer (UICC) and International Federation of Gynaecology and Obstetrics (FIGO) which all rely upon characteristics of primary tumour, nodes and metastases as a basis. The Ann Arbor and Dukes classifications also use similar definitions and principles [4,5].

The TNM system depends on the combination of different characteristics of the tumour. T describes the primary tumour size and/or extent, N describes the presence or absence of regional lymph node metastasis and M describes the presence or absence of distant metastasis. Each of these components is divided into numerical subsets (T0 – T4, N0 – N3, M0 – M1) which describe how advanced the malignancy is. The definitions of these subsets are specific for each tumour and are delineated in a handbook [4]. Depending on the specific combination of T, N and M, an individual cancer will be assigned to a "stage".

For most cancers, the staging of the tumour depends only on TNM. However, some tumours also require additional information for staging (for example, serum tumour markers for testicular cancer) [4]. Additional prognostic factors may be increasingly included in the delineation of the TNM stage grouping. The TNM system is flexible and accepted worldwide for patient care, and has been validated as being relevant to the clinical practice of oncology [6].

Western Australia is the largest state of Australia. With an area of more than 2 500 000 sq km, a 12 500 km coastline and spanning 2 400 km from north to south, it occupies a third of the continent. The total population of Western Australia is just under 1.5 million people of whom 1.2 million live in and around the capital city of Perth.

The Western Australian Cancer Registry (WACR) is a population-based cancer registry, which was established in 1981, based on the mandatory reporting of cancers by pathologists, haematologists and radiation oncologists [7]. The WACR routinely collects data relating to tumour location, type, basis and date of diagnosis, and grade, together with demographic information and some possible sources of further information. At present the WACR does not routinely register information on cancer stage. Recognising the need for these data, this project aimed to determine the feasibility, in terms of ascertainment and effort required, of adding staging data to the WACR.

There are four major public hospitals in Perth and three major private hospitals. Each of the public hospitals has a Hospital Based Cancer Registry (HBCR) which collects information on a limited range of cancer types, including information on staging at the time of diagnosis.

## Methods

The project aimed to collect staging data on a selection of cancer cases from among those notified to the WACR, including cases notified in the past (retrospective data collection) and in the present (prospective data collection). The retrospective cases were those diagnosed in 1998 (300) and in January – June 2002 (150); the prospective cases (150) were those diagnosed after June 2002. The cancer types were selected on the basis of the 20 most common incident cancers in 2000 (apart from non-melanocytic skin cancers, which are not collected by WACR). For each of the five most common cancer types (prostate, colorectal, melanoma, breast and lung cancers) 60 cases were randomly selected for staging and for the remaining 15 cancer types, 20 cases were selected. Approval for access to medical records was requested from each hospital in which selected cases had been treated.

There were 4 sources for collecting data for the staging: the WACR, the HBCRs, hospital medical records, and responses to enquiry letters to treating doctors. For most cancers, this was the order in which the sources were approached. The first step involved reviewing the WACR files and extracting available staging data from pathology reports. If the cancer could not be fully staged from the WACR files, the HBCRs were approached for any staging information they held. If full staging information was not available from WACR or HBCRs, a project officer reviewed casenotes at both private and public hospitals. For reasons related to costs, medical records were not searched at country hospitals and small city hospitals. If the cancer was still unable to be staged, a letter was sent to the treating clinician requesting staging information.

For some cancers, specific local circumstances meant it was more efficient to undertake the steps in a different order, or to omit some steps. Gynaecological cancers (cervix, uterus and ovary), are to a large extent treated in one public hospital with an active HBCR. The FIGO summary stage for all gynaecological cancers was generally the only information used for these cancers. For colorectal cancer, information was obtained initially from the HBCRs and also obtained from the WA Research Tissue Network, which collects statewide data (including stage) on certain cancers. Cases which could not be staged using these resources were then researched using other sources as necessary. For the lymphohaematopoietic malignancies, for which the TNM system is not appropriate, and for brain cancers, WACR files only were reviewed. For melanoma, most cases do not require hospital admission so HBCRs and hospital casenote reviews were not done.

It should be noted that the stage at diagnosis commonly includes data collected over a period of up to 4 months [8] although the WACR uses 3 months as a routine.

We calculated the proportion of cases that could be staged after each step of the staging process overall and stratified by prospective and retrospective groupings.

In order to definitively stage many cancers, considerable clinical investigation is required. For example, lymph nodes need to have been dissected or biopsied, or extensive searches for metastases need to have been undertaken. These further investigations have associated costs and risks and may not always be clinically warranted. For example, if a melanoma is found to be level 1, and there is no clinical evidence of spread, investigations such as chest X-rays and bone scans are unlikely to be performed. In addition, some cases do undergo further investigations but the results are held in private rooms, or medical records do not contain any negative information such as the absence of metastases. In addition, doctors may not have responded to our letters asking for information on stage. For many of the cases in this study, therefore, it was not possible to stage the cancer definitively because of the lack of information on regional nodal status or the presence or absence of distant metastases. Many of these cases are most likely to be early stage cancers.

The data for those cases that were not stageable were further examined, and two different assumptions were applied. The first assumption was applied to cases which had data for T and N but which had no assessment of distant metastasis (MX). The assumption made was that MX was equivalent to M0 (no distant metastases). This is summarized as MX = M0 in the tables.

The second assumption was applied to cases with data for T and M, but with no assessment of regional nodal involvement (NX). The assumption made was that NX was equivalent to N0 (no regional nodal involvement). This is summarized as NX = N0 in the tables.

The number of cases that could be staged if both assumptions were made for the same case (ie NX = N0 and MX = M0) was also calculated. All original data (without assumptions) was stored separately.

The costs of collecting staging data were also estimated. For each cancer type, actual times from the study were extrapolated to costs based on a whole year's collection of staging data. Tasks contributing to this time included: reviewing pathology reports; looking at electronic files at WACR; examining medical case notes; writing enquiry letters and reviewing replies. Added to this were general costs including travel, liaison with data holders, training, leave, general office duties, and delays due to competing priorities of hospital and other non-WACR staff.

## Results

The focus of this project was the process of collecting staging data, and not the actual stages of the cancers.

The main finding from this feasibility study is that staging completeness is very dependent on cancer type (Table 1). Each type has its own issues and complications. However, in order to summarize the results we have grouped some types together as follows:

**Table 1:** Percentage of cases staged, by cancer type and process used. Percentages shown are cumulative, beginning from the left.

Cancer type	**Staged from WACR data alone (%)**	**Staged from WACR and HBCRs, WARTN (%)**	**Staged after all completed steps (no assumptions) (%)**	**Staged after all steps with assumption/s (%)**
**Group A: Could be staged now**				
Ovary	60^	100	100	100
Cervix	16^	95	100	100
Uterus	50^	85	95	95
				
**Group B: Could be staged now, making MX = M0 assumption**				
Breast	0	12	65	95
Colorectal	12^	53	80	92
				
**Group C: Could be staged now, with NX = N0 and MX = M0 assumptions**				
Melanoma	0	(0)	57	100
Prostate	2	5	34	97
				
**Group D: Could be started now with MX = M0, but long term collection requires system changes**				
Stomach**	25	25	70	95
Lung	18	38	76	86
Pancreas	45	45	70	80
Thyroid**	10	10	47	79
Testis**	10	10	75	75
Kidney**	15	20	65	70
				
**Group E: Staging not feasible at present**				
Oesophagus**	0	0	50	65
Bladder**	0	0	40	55
Lip	0	(0)	37	42
Lymphoma	44	(44)	44	44
Myeloma	0	(0)	0	0
Leukaemia	0	(0)	0	0
Brain	0	(0)	0	0

^ These numbers could have been higher as the external databases were searched first, and WACR later searched only for incomplete cases.

** Only one HBCR currently collects data on these cancers except bladder, for which two HBCRs are collecting data.

() Numbers in parentheses indicate that the additional data source/s indicated by the column header, was/were not accessed as they were either not applicable to the cancer type, or research suggested the additional effort would be unrewarding.

• Group A: cancers for which virtually complete staging data could be obtained relatively easily. Relatively complete information on stage of these cancers was available with current systems.

• Group B: cancers for which relatively high proportions of cancers could be fully staged, and for which the assumption MX = M0 allows almost complete staging. If it was considered reasonable to apply this assumption, relatively complete information on stage would be available on these cancers.

• Group C; cancers which can almost all be staged making the assumptions NX = N0 and MX = M0. If it was considered reasonable to apply these assumptions, relatively complete staging information could be collected.

• Group D; cancers for which it is more risky to apply the assumptions. Accurate collection requires system changes in order to obtain better information on stage.

• Group E; cancers for which staging is not feasible at present.

**Group A **cancers (Table 1) consisted of the gynaecological cancers: ovary, cervix and uterus. The use of the HBCR at the gynaecological hospital was clearly crucial for staging these cancers, increasing the stageable proportion of cases from 60% to 100% for ovarian cancer and from 16% to 95% for cervical cancer. For these cancers there was no need to apply any assumptions. Relatively complete staging data could be collected easily.

**Group B **cancers consisted of breast and colorectal cancer. For these two cancers, a reasonable number of cases could be staged fully using the standard 4 steps. For colorectal cancer, 80% of cases could be staged after all four steps were completed, with this increasing to 92% if the assumption MX = M0 was applied. For breast cancer 65% of the cases could be staged after all four steps were completed, increasing to 95% when applying the assumption MX = M0.

**Group C **cancers consisted of melanoma and prostate cancer. Only 34% of prostate cancer cases and 0% of melanoma cases could be staged using only the WACR, mainly because of lack of information on nodal status and metastases. However, if both assumptions of NX = N0 and MX = M0 were applied this increased to 100% of prostate cancers and 97% of melanomas.

**Group D **cancers include cancers of the stomach, lung, pancreas, thyroid, testis and kidney. Cancer types in Group D would require additional clinical input to achieve adequate proportions of staged cancers. Making the assumption of MX = M0, a relatively high proportion of cases may be able to be staged. However, the acceptability of the assumptions differs among cancers.

Cancer types in **Group E **are not able to be staged at present. After all four steps were completed only 50%, 40% and 37% of cases could be staged, for cancers of the oesophagus bladder and lip, respectively. These levels of completeness were still inadequate even after applying the assumption MX = M0. No myeloma or leukaemia cases could be staged at only step 1, and the only lymphoma cases that could be staged were Stage 4. The amount of clinical information required to stage these cancers goes far beyond the pathological details available. There is no accepted staging system for brain cancer.

Costs were estimated assuming that the HBCRs continued to operate. In this case, collection of staging data on Group A cancers would cost approximately 0.1 of a full-time equivalent staff member. Costs of collecting staging data for Groups A and B would require about one half-time staff member and to collect staging data on Groups A, B and C would require about one full-time staff member. This is in relation to a population of 1.5 million.

## Discussion

The main finding from this study is that collection of adequate staging data by population-based cancer registries depends primarily on the type of cancer and the existence of HBCRs or other specialized registries which already collect such data. Because HBCRs already collect high quality staging information in Western Australia, their operation is vital to the efficiency and cost-effectiveness of population-based collection of staging data. As can be seen from the results of this study, in cases where HBCRs covered a large proportion of the cancers seen in WA (for example the gynaecological cancers, or colorectal cancer), the proportion of cases which could be staged was high. In other cases, such as melanoma, for which no HBCR collects staging information, the proportion of cases which could be staged tended to be low. The greater the coverage of HBCRs in terms of cancer types and hospitals, the better the stage collection of the population-based registry can be. While this is cost-efficient for the population based registry, costs have to be borne by the HBCRs.

In this study, we had a short timeline which meant that the data we were able to access from the HBCRs was probably less complete than would be available over a longer period. On the other hand, the fact that we did not collect information from small country hospitals means that we probably slightly overestimated the proportion of cancers which could be staged.

One of the problems in attempting to collect staging data for a variety of different cancer types was that population-based cancer registries such as the WACR usually rely on pathology reports, and do not routinely receive non-pathology reports. These include radiological reports such as CT scans, X-rays and PET scans which contain information on N and M. Other information not routinely supplied to registries may be crucial to staging, such as hormonal assays for testicular cancer, negative lymph node biopsies for breast cancer, and haemoglobin level and cell counts in leukaemia.

Related to this was the necessity to make assumptions about MX and NX. The acceptability of these assumptions needs to be ascertained and is different for the different cancer sites. For example, for melanomas of Clark level I [9], or low Breslow thickness [10], it is probably quite reasonable to assume that NX = N0 and MX = M0. However, for stomach and pancreatic cancer, the viability of the MX = M0 assumption is questionable as these cancers are often at a late stage when diagnosed. This means that cancer sites which had similar proportions of stageable cancers without assumptions were placed in different categories depending on how clinical acceptable the assumptions were. For example, only 54% of the melanomas could be staged without assumptions, and 100% with assumptions and clinically it was thought that the assumptions were reasonable. However, 70% of the stomach cancers could be staged without assumptions, and 95% with assumptions but clinically it was not thought that the assumptions were reasonable.

Where it is not thought appropriate to apply the MX = M0 assumption to all unstaged cancer cases, an alternative strategy would be to work with clinicians in order to develop rules about for which cases it would be appropriate to apply this assumption. A separate study suggests that it is safe to make the MX = M0 assumption in 90% of breast cancer cases with T1, T2 or T3 and either N0 or N1 (personal communication, Padabphet Boutdara) and about 85% of similar colorectal cancer cases [11].

A more difficult problem occurred when the cancer was diagnosed on a biopsy specimen. In these cases, even the information on tumour size (T) was often unable to be ascertained.

Increasing the proportion of cancers which could be staged would require a greater level of input from clinicians than currently exists. For instance, the use of synoptic structured pathology reports would assist the staging of these cancers, by providing a consistent approach to staging, as well as an easy format for the clinicians to provide this information to the cancer registry.

In order to guide our comments on the feasibility of various potential recommendations we interviewed specialist clinicians interested in specific cancer types, to canvass their views on issues relating to staging data collection. Some of the issues raised by clinicians were confidentiality and privacy concerns, and access to and "ownership" of data. In addition, there was disagreement among the clinicians as to whether it was the responsibility of clinicians to do the staging, or whether that should be done by registry staff.

There are several other problems with adding staging to a population based registry. If a formal screening program is introduced there may be better recording of data and less missing information on stage over time. In addition it is important to consider whether staging in a population-based cancer registry is worthwhile if reasonable levels of completeness cannot be achieved. For example, it may be hard to justify collection of data for kidney, testicular, thyroid or pancreatic cancer as 20% – 30% of cases in a statistical analysis would not have staging information.

## Conclusion

Adding stage to a population-based cancer registry is highly dependent on the type of the cancer. However, routinely collected staging is possible for many cancer types with the co-operation of HBCRs and the assumptions of negative nodal or metastatic status for some types. These findings should be generalizable to most cancer registries in developed countries, if hospital-based cancer registries or other specialized databases are accessible.

## Competing interests

The author(s) declare that they have no competing interests.

## Authors' contributions

TT participated in the design of the study, managed the project staff, oversaw the analysis and project report, and assisted with drafting the manuscript.

JW was responsible for the co-ordination of data collection, provided the results for the project report, drafted the project report and edited the manuscript.

PB was responsible for data collection and data entry, conducted the data analyses, contributed to the project report and edited the manuscript.

JH participated in the design of the study, the supervision of the data collection, interpretation of the results, production of the project report and editing the manuscript.

PK oversaw the administration of the grant and edited the manuscript.

HS was Convenor and Chair of the Steering Committee, and participated in editing the manuscript.

LF participated in the design of the study, the supervision of the data collection, interpretation of the results, production of the project report and the drafting of the manuscript
